# Supplementary figures and images for: Real-time denoising of ultrasound images based on deep learning
Source: Med Biol Eng Comput. 2022 Jun 7;60(8):2229–44. doi: 10.1007/s11517-022-02573-5 (PMC9293842; doi:10.1007/s11517-022-02573-5)

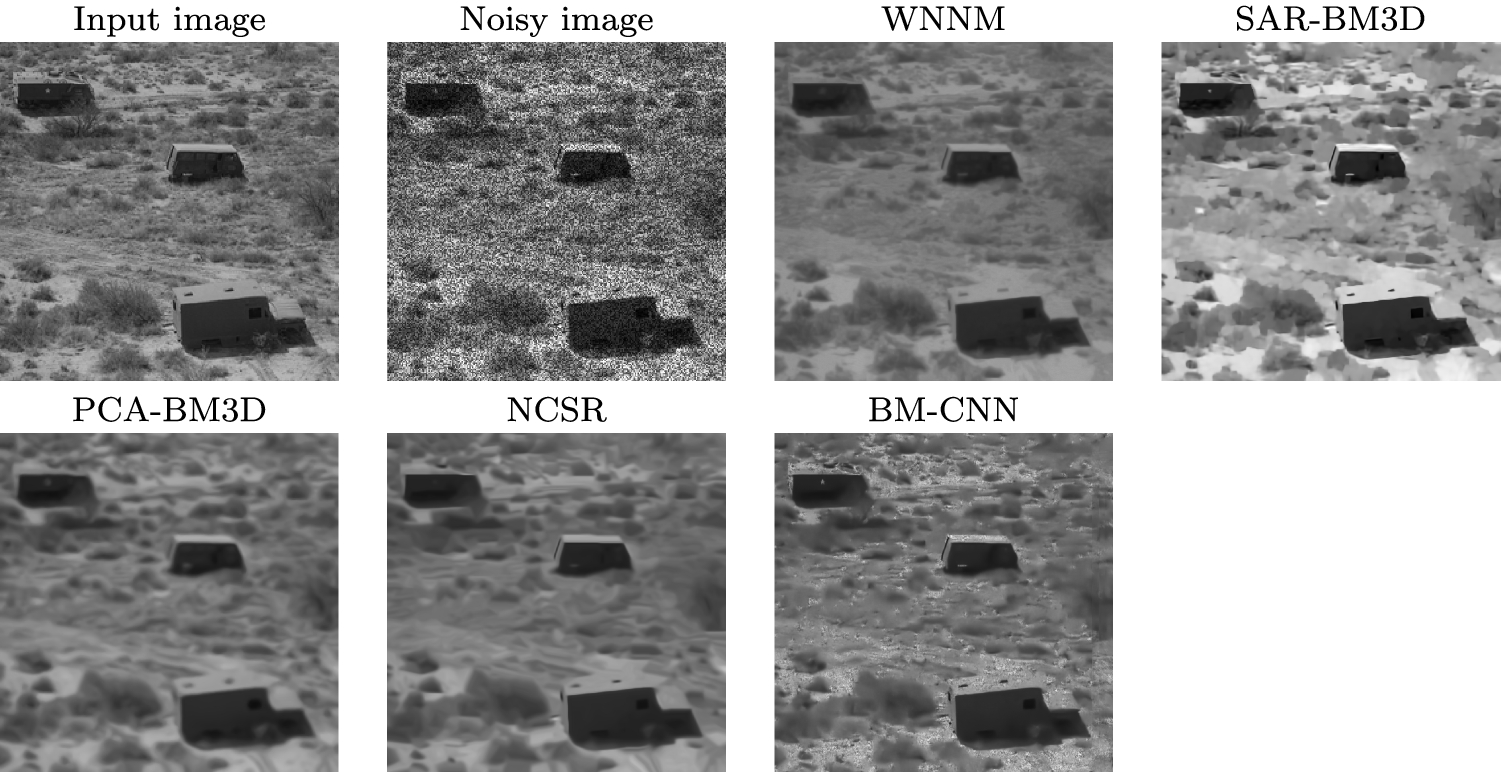

Supplement: Supplementary file 1 — Input (SIPI data set, van image), noisy (speckle noise intensity σ = 0.10), and denoised images. For error metrics, we refer the reader to Table 4 [file 11517_2022_2573_Fig12_HTML.png]

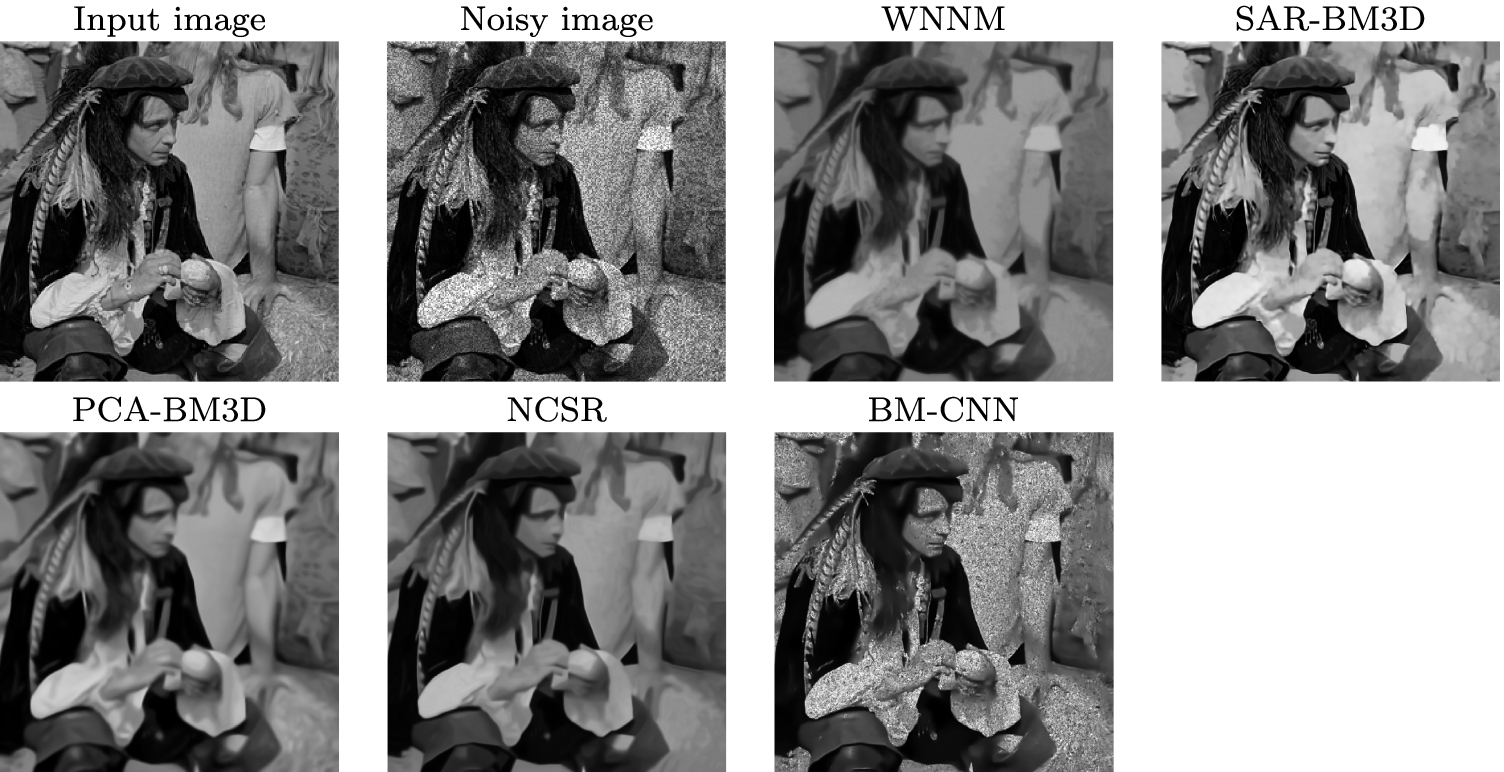

Supplement: Supplementary file 2 — Input (SIPI data set, man image), noisy (speckle noise intensity σ = 0.20), and denoised images. For error metrics, we refer the reader to Table 4 [file 11517_2022_2573_Fig13_HTML.png]

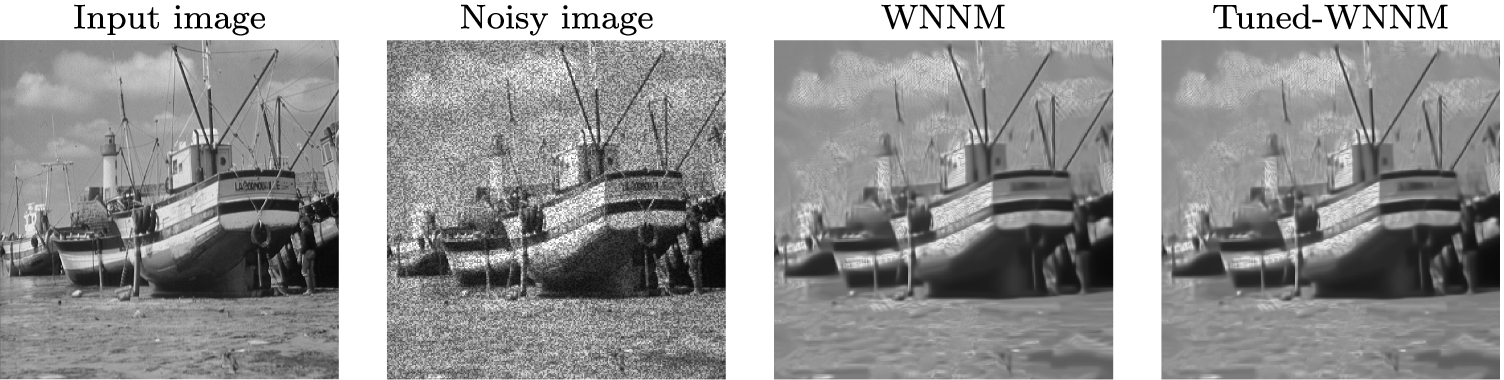

Supplement: Supplementary file 3 — Input (256 × 256), noisy (speckle noise intensity σ = 0.05), and denoised images with WNNM and the tuned-WNNM [file 11517_2022_2573_Fig14_HTML.png]
